# Supplementary material for: Pyruvate Dehydrogenase Kinase 1 inhibition mediated oxidative phosphorylation enhancement in cartilage promotes osteoarthritis progression
Source: BMC Musculoskelet Disord. 2023 Jul 20;24:597. doi: 10.1186/s12891-023-06585-6 (PMC10357736; doi:10.1186/s12891-023-06585-6)
Supplement: Supplementary file 1 — Additional file 1. [file 12891_2023_6585_MOESM1_ESM.zip › MF.Gsea.1653627316242/GOMF_IMMUNOGLOBULIN_RECEPTOR_BINDING.html]

Details for gene set GOMF\_IMMUNOGLOBULIN\_RECEPTOR\_BINDING[GSEA]

|  || Dataset | 55457\_collapsed\_to\_symbols.cls.cls #OA\_versus\_normal.cls.cls #OA\_versus\_normal\_repos |
| Phenotype | cls.cls#OA\_versus\_normal\_repos |
| Upregulated in class | OA |
| GeneSet | GOMF\_IMMUNOGLOBULIN\_RECEPTOR\_BINDING |
| Enrichment Score (ES) | 0.54238856 |
| Normalized Enrichment Score (NES) | 1.8946966 |
| Nominal p-value | 0.0 |
| FDR q-value | 0.022231093 |
| FWER p-Value | 0.283 |
Table: GSEA Results Summary

  

Fig 1: Enrichment plot: GOMF\_IMMUNOGLOBULIN\_RECEPTOR\_BINDING      
 Profile of the Running ES Score & Positions of GeneSet Members on the Rank Ordered List

  

| SYMBOL | TITLE | RANK IN GENE LIST | RANK METRIC SCORE | RUNNING ES | CORE ENRICHMENT || 1 | IGHG1 | immunoglobulin heavy constant gamma 1 (G1m marker) [Source:HGNC Symbol;Acc:HGNC:5525] | 607 | 0.621 | 0.0316 | Yes |
| 2 | IGHV3-72 | immunoglobulin heavy variable 3-72 [Source:HGNC Symbol;Acc:HGNC:5622] | 1062 | 0.504 | 0.0603 | Yes |
| 3 | TRDC | T cell receptor delta constant [Source:HGNC Symbol;Acc:HGNC:12253] | 1100 | 0.496 | 0.1204 | Yes |
| 4 | IGLC2 | immunoglobulin lambda constant 2 [Source:HGNC Symbol;Acc:HGNC:5856] | 1210 | 0.474 | 0.1721 | Yes |
| 5 | IGHA1 | immunoglobulin heavy constant alpha 1 [Source:HGNC Symbol;Acc:HGNC:5478] | 1426 | 0.439 | 0.2111 | Yes |
| 6 | TRBC1 | T cell receptor beta constant 1 [Source:HGNC Symbol;Acc:HGNC:12156] | 1458 | 0.435 | 0.2639 | Yes |
| 7 | IGKC | immunoglobulin kappa constant [Source:HGNC Symbol;Acc:HGNC:5716] | 1477 | 0.432 | 0.3173 | Yes |
| 8 | IGHV3-33 | immunoglobulin heavy variable 3-33 [Source:HGNC Symbol;Acc:HGNC:5596] | 1511 | 0.427 | 0.3690 | Yes |
| 9 | JCHAIN | joining chain of multimeric IgA and IgM [Source:HGNC Symbol;Acc:HGNC:5713] | 1805 | 0.384 | 0.3949 | Yes |
| 10 | IGHM | immunoglobulin heavy constant mu [Source:HGNC Symbol;Acc:HGNC:5541] | 2241 | 0.335 | 0.4036 | Yes |
| 11 | IGHV3-21 | immunoglobulin heavy variable 3-21 [Source:HGNC Symbol;Acc:HGNC:5586] | 2404 | 0.314 | 0.4309 | Yes |
| 12 | IGHV1-69 | immunoglobulin heavy variable 1-69 [Source:HGNC Symbol;Acc:HGNC:5558] | 2461 | 0.307 | 0.4655 | Yes |
| 13 | IGHV4-61 | immunoglobulin heavy variable 4-61 [Source:HGNC Symbol;Acc:HGNC:5655] | 2722 | 0.278 | 0.4806 | Yes |
| 14 | IGHV3-7 | immunoglobulin heavy variable 3-7 [Source:HGNC Symbol;Acc:HGNC:5620] | 2798 | 0.270 | 0.5090 | Yes |
| 15 | IGHV3-73 | immunoglobulin heavy variable 3-73 [Source:HGNC Symbol;Acc:HGNC:5623] | 3082 | 0.241 | 0.5177 | Yes |
| 16 | TRBC2 | T cell receptor beta constant 2 [Source:HGNC Symbol;Acc:HGNC:12157] | 3401 | 0.211 | 0.5198 | Yes |
| 17 | IGHV3-23 | immunoglobulin heavy variable 3-23 [Source:HGNC Symbol;Acc:HGNC:5588] | 3449 | 0.207 | 0.5424 | Yes |
| 18 | IGHV4-34 | immunoglobulin heavy variable 4-34 [Source:HGNC Symbol;Acc:HGNC:5650] | 4746 | 0.091 | 0.4533 | No |
| 19 | IGHV5-51 | immunoglobulin heavy variable 5-51 [Source:HGNC Symbol;Acc:HGNC:5659] | 6399 | -0.035 | 0.3295 | No |
| 20 | FES | "FES proto-oncogene, tyrosine kinase [Source:HGNC Symbol;Acc:HGNC:3657]" | 6491 | -0.042 | 0.3277 | No |
| 21 | IGHV3-20 | immunoglobulin heavy variable 3-20 [Source:HGNC Symbol;Acc:HGNC:5585] | 6859 | -0.068 | 0.3078 | No |
| 22 | IGLL1 | immunoglobulin lambda like polypeptide 1 [Source:HGNC Symbol;Acc:HGNC:5870] | 7544 | -0.122 | 0.2701 | No |
| 23 | FGR | "FGR proto-oncogene, Src family tyrosine kinase [Source:HGNC Symbol;Acc:HGNC:3697]" | 8248 | -0.175 | 0.2377 | No |
| 24 | IGHD | immunoglobulin heavy constant delta [Source:HGNC Symbol;Acc:HGNC:5480] | 9396 | -0.263 | 0.1821 | No |
| 25 | FLNA | filamin A [Source:HGNC Symbol;Acc:HGNC:3754] | 9976 | -0.318 | 0.1775 | No |
| 26 | IGHE | immunoglobulin heavy constant epsilon [Source:HGNC Symbol;Acc:HGNC:5522] | 10676 | -0.390 | 0.1727 | No |
Table: GSEA details [plain text format]

  

Fig 2: GOMF\_IMMUNOGLOBULIN\_RECEPTOR\_BINDING      
 Blue-Pink O' Gram in the Space of the Analyzed GeneSet

  

Fig 3: GOMF\_IMMUNOGLOBULIN\_RECEPTOR\_BINDING: Random ES distribution      
 Gene set null distribution of ES for **GOMF\_IMMUNOGLOBULIN\_RECEPTOR\_BINDING**
